# Natural Plant Sugar Sources of *Anopheles* Mosquitoes Strongly Impact Malaria Transmission Potential

**DOI:** 10.1371/journal.pone.0015996

**Published:** 2011-01-20

**Authors:** Weidong Gu, Günter Müller, Yosef Schlein, Robert J. Novak, John C. Beier

**Affiliations:** 1 Division of Infectious Diseases, School of Medicine, University of Alabama, Birmingham, Alabama, United States of America; 2 Department of Microbiology and Molecular Genetics, Faculty of Medicine, IMRIC, Kuvin Centre for the Study of Infectious and Tropical Diseases, Hebrew University, Jerusalem, Israel; 3 Department of Epidemiology and Public Health, Miller School of Medicine, Center for Global Health Sciences, University of Miami, Miami, Florida, United States of America; Singapore Immunology Network, Agency for Science, Technology and Research (A*STAR), Singapore

## Abstract

An improved knowledge of mosquito life history could strengthen malaria vector control efforts that primarily focus on killing mosquitoes indoors using insecticide treated nets and indoor residual spraying. Natural sugar sources, usually floral nectars of plants, are a primary energy resource for adult mosquitoes but their role in regulating the dynamics of mosquito populations is unclear. To determine how the sugar availability impacts *Anopheles sergentii* populations, mark-release-recapture studies were conducted in two oases in Israel with either absence or presence of the local primary sugar source, flowering *Acacia raddiana* trees. Compared with population estimates from the sugar-rich oasis, *An. sergentii* in the sugar-poor oasis showed smaller population size (37,494 vs. 85,595), lower survival rates (0.72 vs. 0.93), and prolonged gonotrophic cycles (3.33 vs. 2.36 days). The estimated number of females older than the extrinsic incubation period of malaria (10 days) in the sugar rich site was 4 times greater than in the sugar poor site. Sugar feeding detected in mosquito guts in the sugar-rich site was significantly higher (73%) than in the sugar-poor site (48%). In contrast, plant tissue feeding (poor quality sugar source) in the sugar-rich habitat was much less (0.3%) than in the sugar-poor site (30%). More important, the estimated vectorial capacity, a standard measure of malaria transmission potential, was more than 250-fold higher in the sugar-rich oasis than that in the sugar-poor site. Our results convincingly show that the availability of sugar sources in the local environment is a major determinant regulating the dynamics of mosquito populations and their vector potential, suggesting that control interventions targeting sugar-feeding mosquitoes pose a promising tactic for combating transmission of malaria parasites and other pathogens.

## Introduction

With the increased international attention to malaria control and elimination, vector control measures including long-lasting insecticide-treated nets (LLINs) and indoor residual spraying (IRS) play a pivot role in suppressing transmission intensity and disease burden [1], [2]. Nevertheless, scale-up applications of LLINs and IRS inevitably lead to development of insecticide resistance and do not suffice to sustain long-term control effects [3], [4]. There is a need to revisit the life history of mosquitoes to explore innovative control strategies which could synergize with the current intervention tactics [1], [5]. Vector ecology has been reiterated as the key for the development of much-needed new approaches beyond LLINs and IRS for controlling malaria vector species and locally eliminating malaria parasite transmission [6].

The life cycle of female mosquitoes entails foraging behaviors seeking physiologically-required resources such as mates, hosts, resting places, sugar, and oviposition sites. Constrained resources, e.g. food shortage and habitat loss, are the center of concern in animal ecology and conservation biology[7]. In contrast, resources in the life cycle of mosquitoes are conventionally assumed to be ubiquitously available and not to be a limiting factor. Recently, several theoretical studies have examined this assumption and shown that the reduced availability of resources by interventions, e.g., bednets and source reduction of aquatic habitats, can significantly affect population dynamics and the vectorial capacity of pathogen transmitting mosquitoes [8], [9], [10]. However, field evidence is lacking to specifically show how the availability of local resources affects mosquito populations in nature.

Both male and female mosquitoes need sugar, mostly from floral nectar, honeydew and fruits, for nutrition and energy [11]. Although sugar feeding is fundamental for maintaining vital activities of mosquitoes in laboratory, its role in the population dynamics of mosquitoes in nature remains largely unknown. In this study, we describe a natural experiment in which the dynamics of two populations of *Anopheles sergentii* was closely observed in two desert oases in Israel. Malaria was eliminated in Israel during the 1960's [12], [13] but *An. sergentii* remains a major vector of malaria in parts of the Middle East. Specifically, we evaluate the potential impact of the sugar availability on the vectorial capacity of *An. sergentii* by comparison of empirical estimates of abundance, survival rates and the duration of the gonotrophic cycle.

## Materials and Methods

### Ethics Statement

Although the release of *An. sergentii* temporally increased local mosquito populations in the two study sites, the experiment posed no risk of public health because the area had been malaria-free since 1960s and is uninhabited. There were no nomadic people spending the night in any of the oases during the field trial and there are no settlements closer than 20 km to the locations.

### Study area

Field experiments were conducted in two small, uninhabited oases, 5 km apart in the Arava Valley desert environment in Southern Israel. Both sites included small fresh-water springs surrounded by dense non-flowering vegetation in the core of the oasis that became sparser further away from the water. The centers of the two oases with dense vegetation covered an area of about five hectares[14]. Average annual rainfall was 50 to 100 mm, autumn temperatures from 30 to 40°C, and the relative humidity below 50%. At both places, there were herds of camels and donkeys raised by semi-nomadic Bedouin people who occasionally stayed overnight. Other common animals included hares, gazelles, and numerous rodents like sand rats, and gerbils. Riparian plants including *Phragmites australis* (Cav.), A*rundo donax* and tall sedges (Gramineae) were close to the water in the centers, while thickets of desert plants in the periphery were dominated by *Salsola cyclophylla*, *Suaeda fruticosa*, *Atriplex halimus* (Chenopodiaceae) and *Alhagi graecorum* (Papilionaceae). Several *Acacia raddiana* (Mimosaceae) and *Tamarix jordanis* (Tamaricaceae) trees were scattered at different distances from the water. *An. sergentii* breeding in water surrounding the springs was the dominant species accounting for over 80% local mosquitoes. Other mosquito species included *Aedes caspius* and *Culex pipiens* occurring in small percentages[15].

The study was conducted from mid-September to November, 2009. At the time of the experiments, herbaceous undergrowth was grazed out by camels and donkeys with no visible sugar sources like flowering plants and shrubs, fruit, and honeydew in the study areas [16]. The environments of the two oases were very similar except the availability of sugar sources. In one of the oases (hereafter sugar-rich oasis), there were two flowering *A. raddiana* trees which were the preferable source of sugar for the mosquitoes [14]. In contrast, there were no flowering trees in the other oasis (called sugar-poor oasis).

### Mark-release-recapture experiments

To estimate the effects of sugar resource availability on mosquito populations, mark-release-recapture experiments were conducted in the two oases. Released mosquitoes were the F1 generation of field collected *An. sergentii*. Large numbers of blood fed females were collected with UV-CDC traps (Model 1212; John W. Hock, Gainesville, FL, USA) inside four goat tents in the lower Jordan valley near Jericho (about 100 km north of the release areas) in a single night. Under semi-field conditions in the shade of a tent pavilion in the sugar-poor oasis, batches of about 100 females were transferred to cages (80×40×40 cm). Ten 100 ml beakers coated with filter paper, and filled with filtered water from the oasis, then placed in each cage for egg laying. Hatched larvae were reared with Tetramin baby fish food (Tetra Werke, Melle, Germany) in large trays. Groups of 500 newly-emerged mosquitoes were transferred to cylindrical screened paper cartons (20 cm high and 18 cm diameter) and maintained on 5% sugar solution and water through the gauze on the top. The boxes were kept in the shade covered with moist towels.

Newly-emerged mosquitoes collected for two nights were dusted inside the cartons with blue fluorescent powder (Day-Glo fluorescent pigments, Day-Glo Color Co., Cleveland, OH, USA). At the sugar-rich site, marked mosquitoes were released on September 18 in the evening at 20:00 hr. Similarly, yellow-marked mosquitoes were released in the evening of September 22 at the sugar-poor site. At both sites, marked mosquitoes were released at the center of the oasis near the overgrown springs. Mosquitoes that did not leave the cartons by themselves within 15 minutes were recovered, counted, and their numbers were subtracted from the total released.

Mosquito recaptures using CDC UV light traps (model 1212) started two days after the release. Initially, captures were operated on a daily basis for three consecutive days, and then switched to collection at a 2-day interval for the following 45 days (a total of 26 sampling occasions). At each oasis, 12 traps were operated at a distance of around 100 m from the water and the release points of the mosquitoes. The traps were surrounding the inner core of the oases and were hung on tripods, 1 m above the ground. Traps were placed at least 20 m from each other, and in case of the sugar-rich oasis also at least 40 m from the flowering *Acacia* trees. To estimate emigration of released mosquitoes, at each of the two distances (1.1 and 1.7 km) from the east border of the oases, 12 traps were placed about every 50 m apart in semi-circle. Captured mosquitoes were transported alive in cooling bags (around 5°C) to the laboratory, anesthetized, counted, and examined under a stereomicroscope using UV light to identify marked individuals.

### Age grading of mosquitoes

For each sampling occasion (26 in total), physiological ages of subsamples of up to 120 marked and 120 unmarked female mosquitoes were determined by observing the dissected ovarioles (Detinova1962). Ovaries were removed from the body of the mosquito in a drop of PBS under a dissecting microscope. The ovarian sheath was removed with dissecting needles to expose ovarioles for examination of dilatations and pedicels.

### Testing gut contents for sugar and plant tissues

Random samples of marked and unmarked female mosquitoes were tested for sugar feeding using the cold anthrone test for fructose [17], [18] as modified by Schlein & Jacobson [19]. Even the low levels of sugar obtained from plant tissue can produce positive anthrone tests[20]. The reaction solution contained 0.15% anthrone (Sigma, St. Louis, MO, U.S.A.) w/v in 71.7% sulphuric acid. Each mosquito was placed in the well of a microtiter plate and wetted with 20 µl of 100% ethanol. Aliquots of 200 µl reaction solution were added to the wells and the specimens were crushed. After incubation for 60 min at 25°C fructose positive mosquitoes stained the reaction solution blue. Samples of mosquitoes were also tested for plant tissue in the gut using the slightly modified method of Schlein & Müller [21]. For staining, a fresh solution of 0.1% calcofluor (Fluorescent brightener 28, White M2R, CX.I. 40622, Sigma) in 0.45% saline, adjusted to pH 8 with NaOH, was prepared on a weekly basis. Mosquito guts were dissected on microscopic slides in several drops of the solution, mounted on other microscopic slides in a drop of the staining solution and covered with cover slips. Prior to use, all the slides and cover glasses were passed through the flame of a Bunsen burner to eliminate fluorescing particles of paper and cloth. Gut preparations were examined under a phase contrast-fluorescent microscope at a wavelength of 360–440 nm to detect calcofluor-stained cellulose particles [22].

### Data Analyses

The population parameters of the mosquito populations in the two oases were separately estimated to evaluate effects of sugar availability using empirical data of the mark-release-recapture experiments.

### Duration of the gonotrophic cycle

We devised a method below to estimate the length *T_g_* of gonotrophic stage *g* as measured by the number of ovariole dilatations using successive stage-frequency data of recaptures of marked mosquitoes. In contrast to conventional estimates of the gonotrophic cycle of an integer, e.g. 2 - 4 days [23], our estimation was weighted average to take into account the variation of individual mosquitoes. 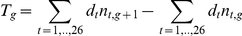



Summation Σ was over 26 sampling sessions. *d_t_* is the number of days of after the release. *n_t,g_* is the number of marked females in *g* gonotrophic stage recaptured on *t* sampling occasion. Then, the mean duration *T* was calculated by averaging all stages except the first and the last because the first gonotrophic cycle for *An. sergentii* is irregular and often requires more than one blood meal [24], and the last category of gonotrophic stage included all combined stages over 10 gonotrophic cycles.

### Estimation of survival rates

To estimate the daily survival rates, we adopted a nonlinear model [25] based on recaptures of marked mosquitoes after a single cohort of release.
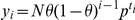
where *N* is the number of marked and released individuals. *θ* is the probability of capture of individual mosquitoes by the sampling method. Note *θ* is different from the empirical recapture rates calculated as the ratio of the total of recaptured marked mosquitoes to the total of released ones. For parameter estimation, least squares method was used using the *nls* function of R statistical package [26].

### Estimation of population size

One of the advantages of the aforementioned nonlinear method [25] is that the capture rate *θ* can be used to estimate population size (*N*) using averaged captures of unmarked mosquitoes (*U*)
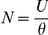



### Estimation of vectorial capacity

From the epidemiological viewpoint, it is important to measure impacts of environmental factors or interventions on the transmission potential of mosquito-borne pathogens. For this purpose, we estimated vectorial capacity (VC) defined as the average number of infectious bites the mosquito could potentially deliver over her lifetime [27]. 
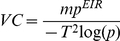



Where *m* was the number of mosquitoes per person. Since there were no people residing in the oases, we assumed the *m* was proportional with estimated population size *N*. Following Dye [28], our focus was on comparison of estimated VC in the two sites rather than calculation of absolute values. For the comparison purpose, *m* in the sugar-poor oasis was arbitrarily set to be 1, and *m* in the sugar-rich oasis was estimated as the ratio of the estimated population size in the sugar-rich oasis to that in sugar-poor site. *T* was the estimated duration of the gonotrophic cycle. *EIP* was the extrinsic incubation period of malaria parasites in mosquitoes which was in a range 10–14 days [23] depending on malaria parasite species and temperature. Here, we adopted a value of 10 days [29] as a conservative estimate to compare the effect of sugar sources on VC because larger values of *EIP* would more drastically amplify the difference in VC between the two oases. Chi-square test was used to assess the differences in frequencies of sugar and plant tissue feeding between mosquitoes in the two oases.

## Results

In the sugar-rich oasis, a total of 26,000 *An. sergentii* mosquitoes (13,950 females and 12,050 males) were released. The average recapture rate was 21% (24 and 18% for females and males, respectively). In the sugar-poor oasis, a total of 32,590 mosquitoes (17,110 females and 15,480 males) were released, with a recapture rate of 5.4% (6.4 and 4.1% for females and males, respectively). The estimated population sizes of local *An. sergentii* were 85,595 and 37,494 in the sugar-rich and sugar-poor sites, respectively (Table 1). Therefore, released mosquitoes accounted for 30 and 87% of the sizes of local populations in the sugar-rich and sugar-poor sites, respectively.

**Table 1:** Estimates of population parameters and malarial vectorial capacity of *Anopheles sergentii* in two oases with different levels of sugar supply (95% confidence intervals are in parentheses).

Parameter	Sugar-poor oasis	Sugar-rich oasis
Gonotrophic duration (days)	3.3(2.1–4.6)	2.4(1.7–3.0)
Survival rate (*p*)	0.72 (0.70–0.74)	0.93 (0.92–0.94)
Individual capture rate (θ)	0.038(0.035–0.042)	0.035 (0.033–0.038)
Population size (N)	37494 (34560–40539)	85595 (79327–92536)
Estimated mosquitoes older than EIP*	6982	29108
Vectorial capacity (VC)	0.024	6.294

*EIP = 10 days

**Figure 1:**
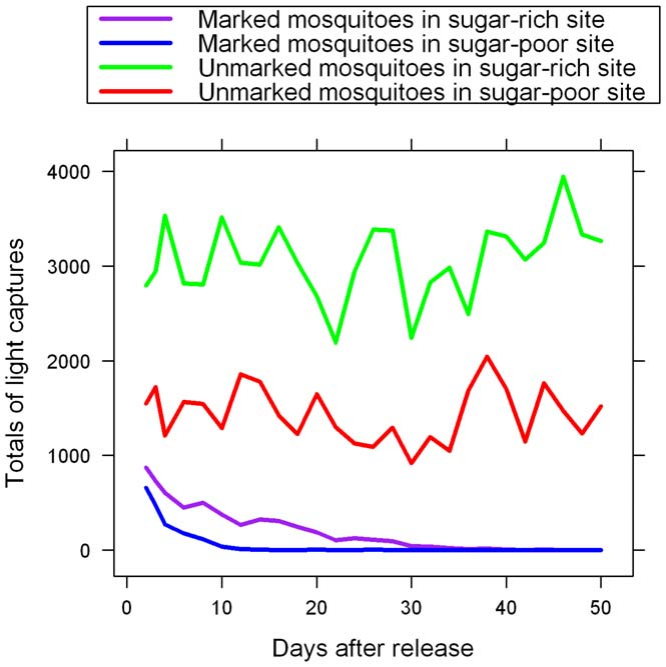
Totals (females + males) of marked and unmarked *Anopheles sergentii* captured in two sites with different levels of sugar supply.

Recaptures of marked mosquitoes in the sugar-poor site declined to a low level quickly following the release, while those in the sugar-rich site gradually decreased over the sampling period (Figure 1). Albeit wide fluctuations, captures of unmarked mosquitoes were centered around their averages (1,437 and 3,059 per day for the sugar-poor and -rich sites, respectively). In the traps placed 1.1 and 1.7 km outside the oases, the recapture rates of marked mosquitoes were 0.35% and 0.23%, respectively, in the sugar-poor oasis compared with 0.10% and 0.05%, respectively, in the sugar-rich oasis. Total captures of unmarked mosquitoes in the traps outside oases were 1222 and 1779 in the sugar-rich and sugar-poor site, respectively.

**Figure 2:**
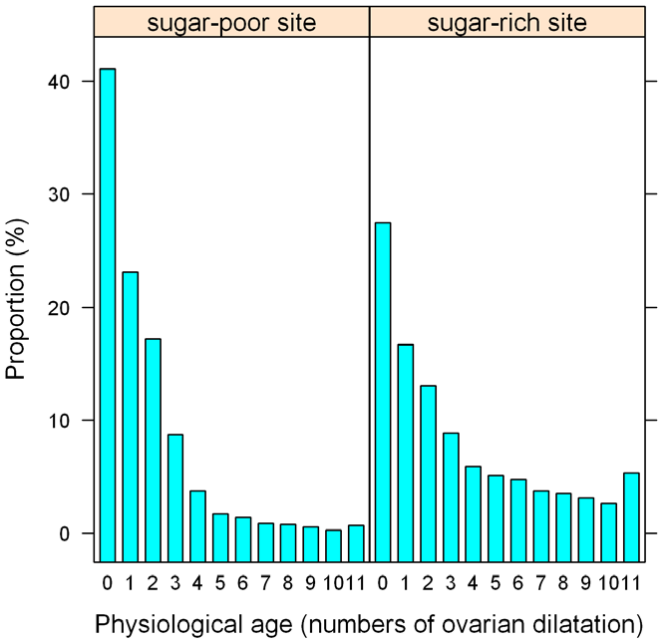
Age structure of unmarked local *Anopheles sergentii*, as determined by the number of dissected ovariole dilatations at two sites with different levels of sugar supply.

Figure 2 shows the physiological age of unmarked mosquitoes determined by age grading methods. A higher proportion (73% of 3,120 females) of parous mosquitoes was detected in the sugar-rich site than that in the sugar-poor site (59% of 3,120 females). In the former site, higher proportions of females exhibited multiple gonotrophic cycles, even >10. The estimated survival rate in the sugar-rich site was 0.93, significantly higher than 0.72 in the sugar-poor site. Therefore, the probability of mosquito survival over the extrinsic incubation (10 days) was ca. 0.04 in the sugar-poor site, as compared to 0.48 in the sugar-rich site.

Anthrone tests showed that 73% (1209/1658) of the mosquitoes from the sugar-rich site had fed on sugar, significantly higher than the 48% (824/1727) from the sugar-poor site (χ^2^ = 223, df = 1, p<0.001). In contrast, plant tissue feeding in the sugar-rich habitat was 0.3% (5/1,710), significantly less than 30% (551/1810) observed in the sugar-poor site (χ^2^ = 599, df = 1, p<0.001).

Mosquitoes in the sugar-rich site averaged 2.36 days per gonotrophic cycle, almost one day shorter (3.3 days) than that in the sugar-poor site (Table 1). Therefore, roughly 4 and 3 gonotrophic cycles were required for the mosquitoes in the sugar-rich and sugar-poor site, respectively; females old enough (10 days) to be capable of malaria parasite transmission represented 34 and 19% of female mosquitoes. The estimated number of capable mosquitoes in the sugar-rich site was 4.2 times of that in the sugar-poor site. Data analysis of recaptures of marked mosquitoes using the nonlinear model revealed significant differences in survival rates and population size between the two sites. Estimates of the individual capture rate were similar between the sampling sites (Table 1), suggesting sampling efficiency of individual mosquitoes did not differ between the sites.

Importantly, the overall impact of sugar availability on malaria vectorial capacity reflects a substantial difference between the two sites. The estimated vectorial capacity in the sugar-rich site was 6.294, 266 times higher than that (0.024) of the sugar-poor site (Table 1).

## Discussion

Our findings demonstrate that the availability of sugar resources in natural environments accounted for over a 250-fold difference in the malarial vectorial capacity of *An. sergentii*, the difference could be more drastic if a larger *EIP* (>10 days) was adopted. In the oasis with the flowering trees of *A. raddiana*, *An. sergentii* exhibited greater population size, higher survival rates and shorter duration of the gonotrophic cycle (more frequent blood feeding and oviposition). The most significant effect of the sugar shortage on vectorial capacity was reduced survival rate, 0.72 vs. 0.93 in the sugar-poor vs. sugar-rich site, because female mosquitoes must be old enough to allow the malaria parasite to develop into sporozoites in the salivary glands to be able to transmit malaria. The probability of mosquito survival over the extrinsic incubation (10 days) was ca. 0.04 in the sugar-poor site, as compared to 0.48 in the sugar-rich site. It is well documented that the effects of interventions on the mosquito survival rates are extremely important [30].

Mark-release-recapture is a commonly used method for estimation of mosquito survival rates in the field. One of the fundamental assumptions is that populations under study are enclosed without migration. However, most of mark-release-recapture studies conducted in natural environments may not meet this requirement. In these situations, declining curves of mosquito recaptures over time reflect at least two distinct processes, i.e., mortality and emigration. In our study, we found only small proportions of released mosquitoes recaptured by outside traps (0.58% and 0.15% in the sugar-poor and-rich sites, respectively). Therefore, the observed declines of recaptures in the two isolated oases were mainly due to mortality. A field study in different habitats in southern Israel over four seasons showed that reductions in availability of sugar sources were related to increased proportions of nulliparous *An. sergentii*, increased mosquito feeding on plant tissues, and poor survival rates [20].

Our previous studies in Israel indicate that female mosquitoes frequently feed on preferred flowering plants, some up to 130 times more attractive than others [14], [31]. In the absence of favorable sugar sources, mosquitoes apparently can switch to sugar-poor plant tissue for sugar [20]. This was the case in the sugar-poor oasis where a 100-fold higher level of plant tissue feeding was observed. Sugar content in these alternative plant tissue sources is frugal, as compared to high concentrations (20–60%) in nectar and honeydew [32], [33], [34]. Evidently, the poor sugar source did not provide sufficient nutrients and energy for maintaining a viable mosquito population in the sugar-poor site.

This study provides evidence that the natural sugar supply could be one of the key determinants driving mosquito populations, reiterating the importance of the availability of resources in mosquito ecology and pathogen transmission. Our recent studies have developed a framework of mosquito foraging for oviposition sites based on biological characteristics, e.g. limited flight ability, short perceptual ranges, and small energetic budget [8], [35]. Under this theory, *Anopheles* mosquitoes only flourish in environments where all resources are available in a range defined by a combination of mosquito flight and perception. In a typical epidemiological setting, distributions of hosts, oviposition sites and sugar sources are heterogeneous. From the viewpoint of foraging mosquitoes, there might be local shortages of certain resources impeding completion of the gonotrophic cycle at focal sites although the resources might not be rare at large scales. Therefore, local shortages of resources might be common in nature, especially for mosquitoes with limited flight and perceptual abilities. In certain environments, e.g. arid or areas with limited numbers of plants and trees, mosquitoes may locally experience sugar shortage.

Observations of frequent sugar feeding in nature and selective feeding on certain plants have led to development of the vector control tactic featured by spraying vegetation with attractive toxic sugar bait (ATSB) or presenting the baits in simple bait stations. Indeed, various successes with this tactic were obtained for decimating local populations of female and male *Culex pipiens*, *Cx. quinquefasciatus*, cistern-dwelling *An. claviger* in peri-urban sites, and *An. sergentii* and *Aedes caspius* in desert oases [14], [31], [36]. For example, a single spray of fermented fruit solutions with 1% (W/V) toxin boric acid on vegetations around larval habitats in Mali, West Africa, obtained 90% reductions in abundances of *An. gambiae* s.l. populations in 30 days (the proportion of older females, i.e., gonotrophic age greater than 3, reduced from 37% to 6%) [36]. It has been noted that intervention strategies targeting sugar feeding in outdoor environments have a great potential to add to ecologically-based IVM for malaria control in Africa [6]. For this purpose, it is fundamental to investigate foraging ecology in nature including the frequency of sugar feeding and preferred plants in local environments. The present study provides evidence that anopheline mosquitoes require natural sugar sources for maintaining viable populations and that these resources could be a limiting factor in regulating population dynamics in certain environments.

## References

[1] WHO (2008). WHO position statement on integrated vector management.. Wkly Epidemiol Rec.

[2] WHO (2007). Insecticide treated mosquito nets: A position statement.. Global Malaria Programme.

[3] Read AF, Lynch PA, Thomas MB (2009). How to make evolution-proof insecticides for malaria control.. PLoS Biol.

[4] Chambers RG, Gupta RK, Ghebreyesus TA (2008). Responding to the challenge to end malaria deaths in Africa.. Lancet.

[5] WHO (2004). Global strategic framework for integrated vector management.. WHO/CDS/CPE/PVC/2004.

[6] Ferguson HM, Dornhaus A, Beeche A, Borgemeister C, Gottlieb M (2010). Ecology: a prerequisite for malaria elimination and eradication.. PLoS Med.

[7] Tracy CR, Nussear KE, Esque TC, Dean-Bradley K, Tracy CR (2006). The importance of physiological ecology in conservation biology.. Integr Comp Biol.

[8] Gu W, Regens JL, Beier JC, Novak RJ (2006). Source reduction of mosquito larval habitats has unexpected consequences on malaria transmission.. Proc Natl Acad Sci U S A.

[9] Killeen GF, McKenzie FE, Foy BD, Bogh C, Beier JC (2001). The availability of potential hosts as a determinant of feeding behaviours and malaria transmission by African mosquito populations.. Trans R Soc Trop Med Hyg.

[10] Killeen GF, Smith TA, Ferguson HM, Mshinda H, Abdulla S (2007). Preventing childhood malaria in Africa by protecting adults from mosquitoes with insecticide-treated nets.. PLoS Med.

[11] Yuval B (1992). The other habit: sugar feeding by mosquitoes.. Bull Soc Vector Ecol.

[12] Farid MA (1956). The implications of Anopheles sergenti for malaria eradication programmes east of the Mediterranean.. Bull Wld Hlth Org.

[13] Zahar AR (1974). Review of the ecology of malaria vectors in the WHO Eastern Mediterranean Region.. Bull Wld Hlth Org.

[14] Müller GC, Schlein Y (2006). Sugar questing mosquitoes in arid areas gather on scarce blossoms that can be used for control.. Int J Parasitol.

[15] Müller GC, Kravchenko VD, Schlein Y (2008). Decline of *Anopheles sergentii* and *Aedes caspius* populations following presentation of attractive toxic (spinosad) sugar bait stations in an oasis.. J Am Mosq Control Assoc.

[16] Zohary M, Orshansky G (1949). Structure and ecology of the vegetation in the Dead Sea region of Palestine.. Palest J Bot.

[17] Van Handel E (1968). Direct microdetermination of sucrose.. Analyt Biochem.

[18] Van Handel E (1972). The detection of nectar in mosquitoes.. Mosq News.

[19] Schlein Y, Jacobson RL (1994). Mortality of *Leishmania major* in *Phlebotomus papatasi* caused by plant feeding of the sandfies.. Am J Trop Med Hyg.

[20] Müller GC, Schlein Y (2005). Plant tissues: the frugal diet of mosquitoes in adverse conditions.. Med Vet Entomol.

[21] Schlein Y, Muller G (1995). Assessment of plant tissue feeding by sand flies (Diptera: Psychodidae) and mosquitoes (Diptera: Culicidae).. J Med Entomol.

[22] Kasten FH, Clark G (1980). Methods for fluorescence microscopy;.

[23] Killeen GF, McKenzie FE, Foy BD, Schieffelin C, Billingsley PF (2000). A simplified model for predicting malaria entomologic inoculation rates based on entomologic and parasitologic parameters relevant to control.. Am J Trop Med Hyg.

[24] Beier MS, Beier JC, Merdan AA, el Sawaf BM, Kadder MA (1987). Laboratory rearing techniques and adult life table parameters for *Anopheles sergentii* from Egypt.. J Am Mosq Control Assoc.

[25] Buonaccorsi JP, Harrington LC, Edman JD (2003). Estimation and comparison of mosquito survival rates with release-recapture-removal data.. J Med Entomol.

[26] Team RDC (2008). R: A language and environment for statistical computing..

[27] Garrett-Jones C, Grab B (1964). The assessment of insecticidal impact on the malaria mosquito's vectorial capacity, from data on the proportion of parous females.. Bull World Health Organization.

[28] Dye C (1986). Vectorial capacity: must we measure all its components?. Parasitol Today.

[29] Gu W, Novak RJ (2005). Habitat-based modeling of impacts of mosquito larval interventions on entomological inoculation rates, incidence, and prevalence of malaria.. Am J Trop Med Hyg.

[30] Smith DL, McKenzie FE (2004). Statics and dynamics of malaria infection in *Anopheles* mosquitoes.. Malar J.

[31] Schlein Y, Muller GC (2008). An approach to mosquito control: using the dominant attraction of flowering *Tamarix jordanis* trees against *Culex pipiens*.. J Med Entomol.

[32] Corbet SA, Willmer PG, Beament JWL, Unwin DM, Prys-Jones OE (1979). Post-secretory determinants of sugar concentration in nectar.. Plant Cell Environ.

[33] Parida AK, Das AB, Sanada Y, Mohanty P (2004). Effects of salinity on biochemical components of the mangrove, Aegiceras corniculatum. Aquat Bot.

[34] Auclair JL (1963). Aphid feeding and nutrition.. Annual Review of Entomology.

[35] Gu W, Novak RJ (2009). Agent-based modelling of mosquito foraging behaviour for malaria control.. Trans R Soc Trop Med Hyg.

[36] Müller GC, Beier JC, Traore SF, Toure MB, Traore MM (2010). Successful field trial of attractive toxic sugar bait (ATSB) plant-spraying methods against malaria vectors in the *Anopheles gambiae* complex in Mali, West Africa.. Malar J.

